# Books, Notices and Book Reviews

**Published:** 1887-10

**Authors:** 


### BOOKS, NOTICES AND BOOK REVIEWS.

THE PRINCIPLES OF THEORETICAL CHEMISTRY, WITH SPECIAL REFERENCE TO THE CONSTITUTION OF CHEMICAL COMPOUNDS. By IRA REMSEN, Professor of Chemistry in the Johns Hopkins University. Third Edition. Enlarged and thoroughly revised. Pp. xi and 318, duodecimo. PHILADELPHIA: LEA BROTHERS & Co. 1887.

The author has undertaken to give a clear and yet simple explanation of the theory of the constitution of chemical compounds. He admits the impossibility of final knowledge in regard to many of the claimed facts of the science of chemistry, and the probability of future changes in what are now believed to be established facts. The book is admirable in every respect, and is especially worthy of the careful attention of physicians.

THE PRINCIPLES OF ANTISEPTIC METHODS APPLIED TO OBSTETRIC PRACTICE. By DR. PAUL BAR. Translated by HENRY D. FREY. Pp. vii and 175. PHILADELPHIA: P. BLAKISTON, SON & Co. CHICAGO: W. T. KEENER. 1887.

There is no evidence in this book which shows when it was written, but it is evidently of recent date, and the author's notes, marked by brackets in the text, make it a reliable guide in its special field. It is systematically arranged and the list of authorites upon which it is based is large. It is a practical book but much larger than the subject matter which it contains demands.

DIFFERENTIAL DIAGNOSIS: A MANUAL OF THE COMPARATIVE SEMEIOLOGY OF THE MORE IMPORTANT DISEASES. By F. DE HAVILLAND HALL, M. D. Third enlarged American edition. Edited by FRANK WOODBURY, M. D. Pp. xi. and 255. PHILADELPHIA: D. G. BRINTON. 1887.

The additions in this edition are principally in the chapter on diseases of the nervous system. In its present form the book will not only retain its many old friends, but it is certain to gain many new ones. It is a most excellent and useful book.

